# Expression of tardigrade disordered proteins impacts the tolerance to biofuels in a model cyanobacterium *Synechocystis sp.* PCC 6803

**DOI:** 10.3389/fmicb.2022.1091502

**Published:** 2023-01-04

**Authors:** Heao Zhang, Qingyang Liu, Qing Liang, Boxiang Wang, Zixi Chen, Jiangxin Wang

**Affiliations:** ^1^Whittle School and Studios, Shenzhen, Guangdong, China; ^2^Shenzhen Link Spider Technology Co., Ltd., Shenzhen, China; ^3^Shenzhen Key Laboratory of Marine Bioresource and Eco-environmental Science, Shenzhen Engineering Laboratory for Marine Algal Biotechnology, Guangdong Provincial Key Laboratory for Plant Epigenetics, College of Life Sciences and Oceanography, Shenzhen University, Shenzhen, China

**Keywords:** tardigrade disordered protein, biofuel, tolerance, RNA-Seq, *Synechocystis*, cytoplasmic abundant heat soluble

## Abstract

Tardigrades, known colloquially as water bears or moss piglets, are diminutive animals capable of surviving many extreme environments, even been exposed to space in low Earth orbit. Recently termed tardigrade disordered proteins (TDPs) include three families as cytoplasmic-(CAHS), secreted-(SAHS), and mitochondrial-abundant heat soluble (MAHS) proteins. How these tiny animals survive these stresses has remained relatively mysterious. Cyanobacteria cast attention as a “microbial factory” to produce biofuels and high-value-added chemicals due to their ability to photosynthesis and CO_2_ sequestration. We explored a lot about biofuel stress and related mechanisms in *Synechocystis sp.* PCC 6803. The previous studies show that CAHS protein heterogenous expression in bacteria, yeast, and human cells increases desiccation tolerance in these hosts. In this study, the expression of three CAHS proteins in cyanobacterium was found to affect the tolerance to biofuels, while the tolerance to Cd^2+^ and Zn^2+^ were slightly affected in several mutants. A quantitative transcriptomics approach was applied to decipher response mechanisms at the transcriptional level further.

## 1. Introduction

Tardigrades, also known as water bears and moss piglets, are diminutive animals capable of surviving many extreme conditions, like very high/low temperatures, radiation, osmotic shock, exposure to chemicals, and very low pressure close to the vacuum of outer space that is harmful to other forms of life (Hesgrove and Boothby, 2020; Arakawa and Numata, 2021; Boothby, 2021). How these tiny animals survive these stresses has remained elusive even though they were noticed about 250 years ago. Recently termed tardigrade disordered proteins (TDPs) included three families as cytoplasmic-(CAHS), secreted-(SAHS), and mitochondrial-(MAHS) heat soluble proteins based on their subcellular locations (Hesgrove and Boothby, 2020). All members from these families are either highly expressed constitutively or significantly induced when under desiccation. Experiments also indicate members of the TDP family in allaying cellular disruption caused by different abiotic stresses (Hesgrove and Boothby, 2020). The “glass transition” hypothesis of CAHS proteins and the connection of water contents of CAHS protein were proposed in the desiccation tolerance of tardigrades (Arakawa and Numata, 2021; Boothby, 2021).

This study primarily focuses on three of the CAHS proteins, including CAHS 106094 (DP1, Swiss-Prot: P0CU52.1), CAHS 77580 (DP7, Swiss-Prot: P0CU43.1), and CAHS 86272 (DP8, Swiss-Prot: P0CU46.1) proteins. DP1 and DP7 proteins are found to form a glass-like matrix that prevents the metabolic failure of tardigrades upon desiccation. At the same time, the expression of DP8 is highly induced during desiccation (Boothby et al., 2017). DP1 and DP7 are cytoplasmic abundant heat-soluble proteins resulting from anhydrobiosis in tardigrades, but their specific mechanisms against anhydrobiosis are still unclear (Arakawa and Numata, 2021; Boothby, 2021). Researchers proposed that the tolerance of anhydrobiosis might be because of the stabilization of CAHS proteins as vitrifying small molecules like sugars but not their direct glass transition. DP8 affects survival slightly under dehydration but does not affect survival under freezing conditions (Boothby, 2021). There is also evidence that the hetero-expression of CAHS proteins increases desiccation tolerance in bacteria and yeast (Boothby et al., 2017), and improves the tolerance of human cells to hyperosmotic conditions (Tanaka et al., 2015). However, there is no data to explore whether these proteins could increase or impact abiotic stresses other than desiccation when expressed in heterologous systems, especially in microbes.

Cyanobacteria cast attention as a “microbial factory” to produce biofuels and high-value chemicals due to their ability to photosynthesis and CO_2_ sequestration (Machado and Atsumi, 2012). However, to make the process economically feasible, one major hurdle to overcome is to improve the low cell tolerance to biofuels. Thus, CAHS proteins were expressed in a model cyanobacterium, *Synechocystis sp.* PCC 6803 (hereafter *Synechocystis*), to explore the possibility of improving the abiotic stress tolerance of cyanobacteria. We explored biofuel stress and related mechanisms in *Synechocystis* (Liu et al., 2012; Qiao et al., 2012; Wang et al., 2012; Tian et al., 2013; Zhu et al., 2013; Chen et al., 2014; Pei et al., 2014; Song et al., 2014; Zhu et al., 2015). In this study, three CAHS proteins were expressed in *Synechocystis* to construct several mutants. The tolerance of the mutants to several heavy metal ions and biofuels were evaluated. To further decipher responses at the transcriptional level of CAHS-expressed mutants, we applied transcriptomic analysis to comprehend the transcriptional responses to biofuels in *Synechocystis sp.* PCC 6803. This study provides a novel direction to utilize different stress proteins for improving the tolerance to high-value bioactive chemicals, including biofuels.

## 2. Materials and methods

### 2.1. *Synechocystis* culture and treatment conditions

*Synechocystis sp.* PCC 6803 and mutants were cultivated in BG-11 medium (pH 7.5) under a continuous light with ~50 μmol photons m^−1^ s^−1^ at 30°C. Cell density was measured using an Epoch2 Microplate Reader (BioTek, Winooski, VT, United States) at OD_750_. Mutants harboring the pCB-SC101 plasmid with the SpecR (aminoglycoside adenylyltransferase) gene were maintained in BG-11 medium with 20 mg/ml spectinomycin.

To test the sensitivity of the mutants to environmental stress, five metal ions, and two biofuels were added to the BG-11 medium with the following additional concentrations: 5 μM Cd^2+^, 8 μM Co^2+^, 7 μM Zn^2+^, 2 μM Cu^2+^, 180 μM Mn^2+^, 1.5% (v/v) ethanol, and 0.25% (v/v) 1-butanol. Fresh cultures of mutants were adjusted to OD_750_ = 0.1 and were further diluted to OD_750_ = 0.01, 0.001, and 0.0001. Then, 2.5 μl of the diluted cultures were carefully dropped on the BG-11 agar plates, with the metal ions or biofuels added when preparing the agar plate. After about 1–2 weeks of cultivation, the plates were taken to evaluate the mutants’ sensitivity to the stress conditions.

To measure the growth curve of mutants in liquid BG-11 medium, 2 ml of the *Synechocystis* cells with the initial OD_750_ = 0.1 were cultivated in a 24-well microplate, with ethanol or 1-butanol added as stress, and cell density was measured daily using an Epoch2 Microplate Reader (BioTek, Winooski, VT, United States) at OD_750_. After cultivation for 3 days, the *Synechocystis* cells were collected, frozen in liquid nitrogen, and used for transcriptomic library preparation.

### 2.2. Construction of the *Synechocystis* mutants

For the expression of the three disordered proteins in *Synechocystis*, the DNA sequences of these proteins were optimized according to the codon usage of *Synechocystis* (Supplementary File 1) and fully synthesized by BGI (BGI, Shenzhen, China). The shuttle vector pCB-SC101 (Liu and Pakrasi, 2018) was generated in our laboratory using Gibson assembly according to the reference (Liu and Pakrasi, 2018). Two constitutive promoters were cloned from the upstream of *ssl0452* (*nblA1*) and *sll1321* (*atpA*), and assembled to the pCB-SC101 vector using Gibson assembly, together with the coding sequences of the three disordered proteins (DP1, 7, and 8, linked to promoters as 0452 and 1321), and transformed to *E. coli* DH5α. As previously described (Wang et al., 2016; Bi et al., 2018), the plasmids were isolated and transformed into the WT using electro-transformation. Positive clones were cultivated on BG-11 agar plates with 10 mg/ml spectinomycin for about 2 weeks and were confirmed with colony PCR analysis (mutants referred to 0452DPx and 1321DPx).

### 2.3. RNA isolation, quantification, and qualification

Total RNA was extracted using a Quick-RNA Miniprep Kit (Zymo Research, CA, United States). The isolated RNA of each sample was quantified by NanoDrop (Thermo Fisher Scientific Inc., CA, United States). The degradation and contamination of RNA were monitored with 1% agarose gels. RNA integrity was assessed using the Bioanalyzer 2100 system with the RNA Nano 6000 Assay Kit (Agilent Technologies, CA, United States).

### 2.4. Library preparation for transcriptomic sequencing

Total RNA was used as the input for the transcriptomic sequencing library preparations. mRNA was purified from total RNA with probes (Ribo-Zero rRNA Removal Kit, Illumia, CA, United States) to remove rRNA for further sequencing. Fragmentation was performed with the existence of divalent cations under elevated temperature in the First Strand Synthesis Reaction Buffer (5X). First-strand cDNA was synthesized with random hexamer primer and M-MuLV Reverse Transcriptase, then RNaseH was added to degrade the RNA. The second strand of cDNA was synthesized with DNA polymerase I, while dUTP was used to replace the dTTP in dNTP. After converting the overhangs into blunt ends *via* exonuclease/polymerase activities and the adenylation of 3′ ends of DNA fragments, the adaptors were ligated for downstream hybridization. For constructing a strand-specific library, the second strand of cDNA containing U was degraded with the USER Enzyme. The library fragments were purified with the AMPure XP system (Beckman Coulter, Beverly, United States) to select cDNA fragments with ~370–420 bp in length. After the PCR reaction with the High-Fidelity DNA polymerase, the PCR products were purified with the AMPure XP system (Beckman Coulter, Beverly, United States). Finally, the library quality was assessed on the Agilent Bioanalyzer 2100 system (Agilent Technologies, CA, United States).

### 2.5. Clustering and sequencing

The index-coded samples were clustered on a cBot Cluster Generation System with the TruSeq PE Cluster Kit v3-cBot-HS (Illumia, CA, United States) according to the manufacturer’s instructions. The generated library preparations were sequenced on an Illumina Novaseq platform, and 150 bp paired-end reads were generated (Novogene Co., Ltd., Beijing, China).

### 2.6. Transcriptomic data analysis

Raw data were firstly processed through in-house Perl/bash scripts to remove reads containing adapters, reads containing N base, and low-quality reads to generate clean data. Meanwhile, the clean data’s Q20, Q30, and GC content were calculated. Clean data was aligned to the reference genome (GCF_000009725.1) using Bowtie2 (v2.2.3). The reads numbers mapped to each gene were counted using HTSeq (v0.6.1). The FPKM of each gene was calculated based on the length of the gene and read counts mapped to this gene.

Before differential gene expression analysis, the read counts were normalized by edgeR R package through one scaling normalized factor for each sequenced library. The edgeR R package was used for differential expression analysis. Genes with foldchange >2 and value of *p* < 0.05 were assigned as differentially expressed genes (DEGs).

GOseq R package was used for the Gene Ontology (GO) enrichment analysis of differentially expressed genes. KOBAS software was used to test the statistical enrichment of differential expression genes in the KEGG database. The enriched GO and KEGG terms with value of *p* < 0.05 were considered significant. Terms significantly enriched in at least one comparison were included and visualized for data interpretation, even if they were not significantly enriched in other comparisons. Data visualization was achieved using in-house R scripts.

## 3. Results and discussion

### 3.1. Construction and transformation of the TDP plasmids

**Figure 1:**
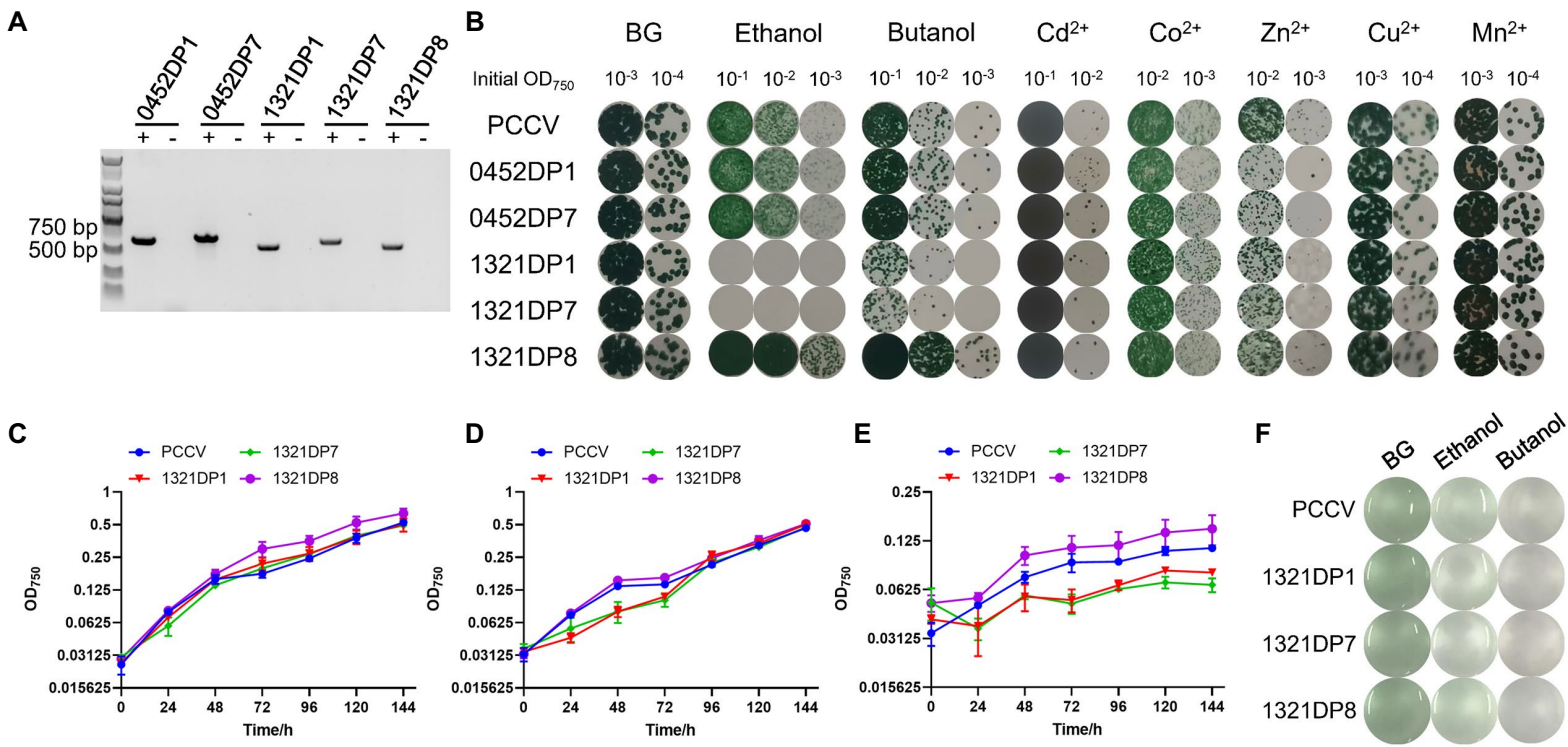
Construction and characterization of the DP mutants. **(A)** Validation of the five DP mutants by PCR. The expected amplicon size: 652 bp (0452DP1), 714 bp (0452DP7), 597 bp (1321DP1), 659 bp (1321DP7), and 585 bp (1321DP8). The symbol “+” means using the corresponding DP mutants as templates, while the symbol “−” means using PCCV as negative control. **(B)** Assays of the PCCV and the five DP mutants under different stress conditions on agar plates. Cultures were adjusted to different concentrations and cultivated on agar plates with different stress conditions. **(C)** Growth curves of PCCV, 1321DP1, 1321DP7, and 1321DP8 in normal BG-11 medium. **(D)** Growth curves of PCCV, 1321DP1, 1321DP7, and 1321DP8 in normal BG-11 medium with 1.5% (v/v) ethanol. **(E)** Growth curves of PCCV, 1321DP1, 1321DP7, and 1321DP8 in normal BG-11 medium with 0.25% (v/v) 1-butanol. **(F)** PCCV, 1321DP1, 1321DP7, and 1321DP8 in normal BG-11 medium, with 1.5% (v/v) ethanol, and 0.25% (v/v) 1-butanol after 48 h cultivation. The error bar represents the calculated standard deviation of the measurements of three biological replicates.

The pCB-SC101 vector was derived from the endogenous plasmid pCB2.4 in *Synechocystis* (Liu and Pakrasi, 2018). All mutants constructed and primers used in this work are listed in Table 1. After successfully constructing all the TDP plasmids in *E. coli*, the plasmids were transformed into the WT by electro-transformation, including the empty pCB-SC101 plasmid as a control. During the experiment, no positive clones were obtained for 0452DP8 even after several times of transformation experiments. According to the reference, the two promoters used here were all constitutive promoters with mild expression levels (Liu and Pakrasi, 2018). They were selected for expressing TDPs to avoid possible toxicity to *Synechocystis*. As P_ssl0452_ is stronger than P_sll1321_, it is possible that the expression of DP8 under P_ssl0452_ has toxicity and caused the failure of obtaining the 0452DP8 mutants. Finally, five TDP mutants were obtained and verified with PCR with 600–800 bp PCR products (Figure 1A) and used for downstream experiments with the engineered strain harboring the empty pCB-SC101 plasmid as a control (named as PCCV).

**Table 1:** *Synechocystis* strains and primers used in this work.

Strain names	Strain descriptions	Strain sources
WT	The wildtype of *Synechocystis sp.* PCC 6803	Our lab
PCCV	Transformed with the empty pCB-SC101 plasmid, used as the control strain	This work
0452DP1	Transformed with the pCB-SC101 plasmid harboring P_ssl0452_ and disordered protein 106,094	This work
0452DP7	Transformed with the pCB-SC101 plasmid harboring P_ssl0452_ and disordered protein 77,580	This work
1321DP1	Transformed with the pCB-SC101 plasmid harboring P_sll1321_ and disordered protein 106,094	This work
1321DP7	Transformed with the pCB-SC101 plasmid harboring P_sll1321_ and disordered protein 77,580	This work
1321DP8	Transformed with the pCB-SC101 plasmid harboring P_sll1321_ and disordered protein 86,272	This work
**Primer names**	**Primer sequences (5’ to 3’)**	**Primer usage**
p0452-F	TCTTACTGTCCCTAGTGCTTGGAACGCAATGACCCAATAACTCGTACTG	Clone the promoter of ssl0452 with homology sequence to pCB-SC101 vector for Gibson assembly
p0452-R-DP1	TATTCATATTCATGGCCTCCATCTAGGTTGCCCTCCAAGGCGACTA	Clone the promoter of *ssl0452* with homology sequence to the disordered protein 106,094 for Gibson assembly
p0452-R-DP7	TGGATTCTTGCTGATAGTTACTCATCTAGGTTGCCCTCCAAGGCGACTA	Clone the promoter of *ssl0452* with homology sequence to the disordered protein 77,580 for Gibson assembly
p0452-R-DP8	CTTCTCATATTGCTGCGACATCTAGGTTGCCCTCCAAGGCGACTA	Clone the promoter of *ssl0452* with homology sequence to the disordered protein 86,272 for Gibson assembly
p1321-F	TCTTACTGTCCCTAGTGCTTGGAACGGGAGAATTGGGGGGAAGAACCAT	Clone the promoter of sll1321 with homology sequence to pCB-SC101 vector for Gibson assembly
p1321-R-DP1	TATTCATATTCATGGCCTCCATCTAGTGACTACTAGCAAGGTGAGA	Clone the promoter of *sll1321* with homology sequence to the disordered protein 106,094 for Gibson assembly
p1321-R-DP7	TGGATTCTTGCTGATAGTTACTCATCTAGTGACTACTAGCAAGGTGAGA	Clone the promoter of *sll1321* with homology sequence to the disordered protein 77,580 for Gibson assembly
p1321-R-DP8	CTTCTCATATTGCTGCGACATCTAGTGACTACTAGCAAGGTGAGA	Clone the promoter of *sll1321* with homology sequence to the disordered protein 86,272 for Gibson assembly
DP1-F	ATGGAGGCCATGAATATGAATA	Clone the disordered protein 106,094 for Gibson assembly and colony PCR to verify the disordered protein 106,094
DP1-R	TGATGCCTGGCTCTAGTATCTCGCGTTTCATCTCGCGGTTACGTT	Clone the disordered protein 106,094 with homology sequence to pCB-SC101 vector for Gibson assembly
DP1-M	CACGTGTTTTGGCTGCGTAA	Colony PCR to verify the disordered protein 106,094
DP7-F	ATGAGTAACTATCAGCAAGAATCCA	Clone the disordered protein 77,580 for Gibson assembly and colony PCR to verify the disordered protein 77,580
DP7-R	TGATGCCTGGCTCTAGTATCTCGTTATTGGTCTGGAACTTTTCACT	Clone the disordered protein 77,580 with homology sequence to pCB-SC101 vector for Gibson assembly
DP7-M	TTGCGATAGGCCTCGGTTTT	Colony PCR to verify the disordered protein 77,580
DP8-F	ATGTCGCAGCAATATGAGAAG	Clone the disordered protein 86,272 for Gibson assembly and colony PCR to verify the disordered protein 86,272
DP8-R	TGATGCCTGGCTCTAGTATCTCAAAAAGGGACTTTATCTTCTCGC	Clone the disordered protein 86,272 with homology sequence to pCB-SC101 vector for Gibson assembly
DP8-M	TGGTCCTTGAAGGCCTGTTC	Colony PCR to verify the disordered protein 86,272
pCB-F	GAGATACTAGAGCCAGGCATCA	Amplify the pCB-SC101 vector for Gibson assembly
pCB-R	CCAAGCACTAGGGACAGTAAGA	Amplify the pCB-SC101 vector for Gibson assembly

Then underlined are overlapping sequences that were used for the Gibson assembly.

### 3.2. Stress response of *Synechocystis* mutants under multiple abiotic stress conditions

An assay with BG-11 agar plates was used to effectively test the response of the five mutants under several stress conditions. For the five metal ion conditions, no significant differences were observed for Cd^2+^, Co^2+^, Cu^2+^, and Mn^2+^ except for the tolerance towards Cd^2+^ of 0452DP1 was slightly improved, while all the engineered mutants were found to be slightly sensitive to Zn^2+^ compared to PCCV (Figure 1B). No significant differences were observed for the ethanol and butanol conditions for 0452DP1 and 0452DP7. However, 1321DP1 and 1321DP7 were significantly more sensitive to selected biofuels. In contrast, 1321DP8 was more tolerant to two biofuels than PCCV (Figure 1B).

Tardigrades are dramatical for surviving rough environments such as extreme high or low temperatures and desiccation. The mechanisms behind unusual tolerance to these harsh stresses remain elusive (Chavali et al., 2017; Janis et al., 2018; Hesgrove and Boothby, 2020; Yagi-Utsumi et al., 2021; Crilly et al., 2022; Malki et al., 2022; Tanaka et al., 2022). Expression of TDP proteins increases desiccation and hyperosmotic tolerance in bacteria, yeast, and human cells (Tanaka et al., 2015; Boothby et al., 2017). Experiments indicate functional roles for TDP family members in resistance against various abiotic stresses (Hesgrove and Boothby, 2020), with various aspects of TDPs for desiccation and other tolerance, suggesting that different members of TDPs may have distinct roles in resistance against different stresses, acting as diverse stress effectors (Koshland and Tapia, 2019). In this study, DP1, DP7, and DP8 showed different roles in defending biofuel stress in cyanobacterial cells, suggesting various defensive roles of different TDPs.

There are several hypotheses for TDP functions and mechanisms. TDPs were proposed to protect cells by forming higher-order assemblies such as reversible aggregates or granules under different stress stimuli (Chavali et al., 2017). Some physiochemical properties suggest that TDP folding may be determined by many physical and chemical conditions such as electrostatic interactions, charge patterning, and expanded conformations (Crilly et al., 2022), and TDPs may experience liquid–liquid phase separations when environmental stresses happen (Janis et al., 2018). In this study, All TDP expressed mutants did not show any tolerance under heavy metal stress conditions, and no significant differences were observed for biofuel conditions in 0452DP1 and 0452DP7. In contrast, 1321DP1 and 1321DP7 were significantly more sensitive to selected biofuels, and only DP8 improved tolerance to two biofuels compared with PCCV. Our data suggest that TDPs may not help defend against heavy metal stress instead of some other abiotic stresses like biofuel. We propose that different stresses cause different cellular responses and result in different TDP folding and phase separations.

Based on the sequences and domain comparison of three TDPs, they all showed a “Disordered-Coiled coil-CAHS motif 1-CAHS motif 2-Disordered” structure, with different positions of the first disordered domain between DP8 and DP1-DP7 (Table 2). At the same time, DP1 and DP7 shared very similar patterns (Table 2). This structural difference of the N-terminal may contribute to the different biofuel tolerance effects of the expression of DP8 in *Synechocystis.*

**Table 2:** Positions of family and domains section of CAHS proteins, DB1, 7, and 8, in this study.

	Disordered	Coiled coil	CAHS motif 1	CAHS motif 2	Disordered
DP1	1–28	90–140	122–140	159–177	198–227
DP7	1–38	83–191	122–140	159–177	200–224
DP8	96–125	115–193	124–142	161–179	204–237

This section provides information on sequence similarities with other proteins and the domain(s) present in a protein, based on https://www.uniprot.org/.

Structures, especially the C-terminal of TDPs, determined their defensive functions. Researchers found that disordered CAHS1 proteins formed stable homo-oligomers *via* the C-terminal α-helical region and became a hydrogel against the desiccation tolerance (Yagi-Utsumi et al., 2021). Similarly, CAHS-8 oligomerizes to long fibers and gels constituted of fibers in a temperature-dependent manner with the helical domain as the core of the fibrillar structure (Malki et al., 2022). Thus, it could be concluded that conserved helical C-terminal is necessary and sufficient for filament formation and gel-transition in a stress-dependent manner by CAHS proteins as the stable cell integrity under dehydration (Tanaka et al., 2022). Besides the conserved C-terminal regions in all selected TDPs in this study, our findings also indicate that N-terminal may also contribute protective functions in abiotic stresses in cyanobacteria.

Even though we did not check desiccation tolerance in the *Synechocystis* strain, some insights could still be obtained from comparison. The disaccharide trehalose was considered to play a functional role in desiccation tolerance in many species (Boothby et al., 2017). Desiccation tolerance studies in yeast, worms, and tardigrades demonstrate that both TDPs and trehalose are the major stress effectors of desiccation tolerance (Koshland and Tapia, 2019). However, trehalose is rare or not detectable in some tardigrades, suggesting different mechanisms tardigrades to survive from desiccation by producing TDPs (Boothby, 2021). Similarly, trehalose cannot be synthesized by *Synechocystis* (Mikkat et al., 1997), and salt-adapted *Synechocystis* cells mainly accumulate glucosyl glycerol (GG) and sucrose (Reed and Stewart, 1985; Hagemann et al., 1997). Thus, further elaborately designed desiccation tolerance experiments should be conducted to explore the possibility of TDPs desiccation tolerance in cyanobacteria.

### 3.3. Growth curve of *Synechocystis* mutants under biofuel stress

The sensitivity to biofuels of the three mutants with the P_sll1321_ promoter was further confirmed with 24-well microplates. No significant differences were observed between the strains without stress (Figure 1C). Under ethanol stress, 1321DP8 showed no differences compared with PCCV, while 1321DP1 and 1321DP7 grew slower during the first 3 days, and no significant differences were found after the fourth day (Figure 1D; Supplementary Table 1). Under butanol stress, all three mutants and the PCCV control grew slowly, but 1321DP8 exhibited better tolerance than the others (Figure 1E; Supplementary Table 1). Since the biomass of cultures under butanol was very low (Figure 1F), cells cultivated under ethanol stress were collected and used for further RNA-seq.

To be noticed, slightly different phenotypes under biofuel stress were found between the results on agar plates and microplates. For example, no growth was found for 1321DP1 and 1321DP7 under ethanol agar plate, but these two mutants only grew slower during the first 3 days. The difference may be caused by different growth micro-environment of cyanobacteria. On the agar plate, not all microbes attached to the agar surface, while in the liquid medium, each cell is exposed to the microenvironments, such as heavy metals, biofuels, or salinity. The cell–cell attachments and close interactions also might be one of possible causes for the difference. Similar difference between agar plate and liquid medium were also reported in previous experiments in *Synechocystis* (Lamb and Hohmann-Marriott, 2017; Agostoni et al., 2018; Wu et al., 2020).

### 3.4. RNA-seq results of *Synechocystis* mutants under ethanol stress

**Figure 2:**
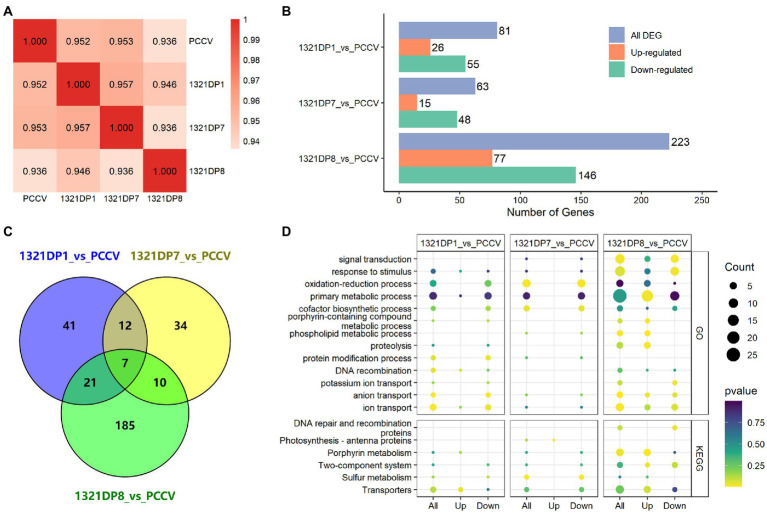
RNA-seq results analysis. **(A)** Pearson correlation analysis between samples. **(B)** Number of DEGs in three mutants. **(C)** Venn diagram of the DEGs in three mutants. **(D)** Pathway enrichment analysis results using DEGs.

To further reveal the differences among several *Synechocystis* mutants, four samples cultivated under ethanol stress were collected and used for transcriptomic data collection. After data trimming, over 1 GB of high-quality clean data was obtained for each sample. The clean data was then aligned to the reference genome, resulting in over 83% unique map rate for all samples (Table 3). After quantification of the expression for all the genes, the Pearson correlation coefficients between samples were calculated using the FPKM values, and 1321DP8 was found to have the lowest coefficients with other samples (Figure 2A). The quantification results confirmed the expression of TDPs in the mutants (Supplementary Table 2). The readcount values of TDPs in 1321DP1 and 1321DP8 were higher than the readcount values of *sll1321*. In 1321DP7, although only 137 reads were mapped to DP7, the FPKM value of DP7 was near 50, suggesting the expression of DP7 in 1321DP7. Statistics of DEGs also showed when compared with PCCV, 1321DP8 has more DEGs than the other two DP mutants (Figure 2B), and over 80% of the DEGs in 1321DP8 were uniquely detected (Figure 2C). In addition, the number of down-regulated DEGs was more than the number of up-regulated DEGs in all three DP mutants (Figure 2B).

**Table 3:** Statistics of RNA-seq data.

	PCCV	1321DP1	1321DP7	1321DP8
Raw reads	7,719,810	7,941,990	8,115,158	7,615,144
Clean reads	7,632,440	7,881,858	8,052,880	7,558,308
Raw bases	1.16G	1.2G	1.22G	1.15G
Clean bases	1.15G	1.19G	1.21G	1.14G
Error rate	0.03	0.03	0.03	0.03
Q20	97.65	97.75	97.74	97.82
Q30	93.24	93.35	93.19	93.39
GC content	49.97	50.37	50.23	50.3
rRNA	0.47	0.44	0.38	0.39
Total mapped	7,366,806 (96.52%)	7,838,479 (99.45%)	7,941,202 (98.61%)	7,509,040 (99.35%)
Multiple mapped	958,925 (12.56%)	1,258,513 (15.97%)	1,182,923 (14.69%)	1,125,455 (14.89%)
Uniquely mapped	6,407,881 (83.96%)	6,579,966 (83.48%)	6,758,279 (83.92%)	6,383,585 (84.46%)
Read-1 mapped	3,206,528 (42.01%)	3,292,885 (41.78%)	3,381,947 (42%)	3,194,280 (42.26%)
Read-2 mapped	3,201,353 (41.94%)	3,287,081 (41.7%)	3,376,332 (41.93%)	3,189,305 (42.2%)
Reads map to ‘+’	3,204,036 (41.98%)	3,290,121 (41.74%)	3,379,175 (41.96%)	3,192,180 (42.23%)
Reads map to ‘−’	3,203,845 (41.98%)	3,289,845 (41.74%)	3,379,104 (41.96%)	3,191,405 (42.22%)
Reads mapped in proper pairs	6,343,264 (83.11%)	6,515,694 (82.67%)	6,698,486 (83.18%)	6,323,232 (83.66%)

The detected DEGs in the three DP mutants were used for GO and KEGG enrichment analysis, respectively, and the results were integrated and visualized for further analysis (Figure 2D). The result showed 1321DP1 down-regulated pathways involved ion and anion transport, protein modification, primary metabolic and oxidation-reduction processes, cofactor biosynthetic process, and response to stimulus in GO enrichment analysis. 1321DP7 showed similar responses with 1321DP1: with almost down-regulated processes and pathways, such as ion and anion transport, primary metabolic and oxidation–reduction processes, and cofactor biosynthetic process in GO enrichment analysis.

*Synechocystis* cells tend to up-regulate specific transporters and efflux pumps to eliminate the toxicity when suffering biofuel stresses. The up-regulation can be mediated by Two-component signal transduction systems (TCSs; Fu et al., 2016). As expected, sulfur metabolism and transporters pathways were KEGG enriched in 1321DP1 and 1321DP7 mutant cells, suggesting the DP1 and DP7 also trigger a similar ethanol response in cyanobacteria.

The previous RNA-seq and proteomic results showed that ethanol exposure induced genes involved in many stress responses, like heat shock proteins, transporters, as well as cell envelope modifiers, as a complicated cellular response (Wang et al., 2012), consistent with our observation in the cells upon ethanol exposure. Similarly, GO and KEGG analyses showed that many transporting, membrane-bound, oxidative stress, and sulfur relay system and photosynthesis-related proteins were induced against another biofuel hexane (Liu et al., 2012). Compared with DP1 and DP7, the expression of DP8 in cyanobacteria involved more processes and pathways. In GO enrichment analysis, more processes were up-regulated in 1321DP8 mutant cells, including signal transduction, response to stimulus, porphyrin-containing compound metabolic, phospholipid metabolic, and proteolysis. Based on KEGG enrichment, porphyrin metabolism and TCSs were significant in 1321DP8 mutant cells. Previous OMICS analyses revealed some TCS genes induced by biofuel stresses in *Synechocystis* (Fu et al., 2016), and knock-out of a TCS *slr1037* increases *Synechocystis* sensitivity to butanol (Fu et al., 2016). Compared with DP1 and DP7, DP8 might involve more in TCS for more effective tolerance against ethanol stress.

Based on the fold changes, we identified the top 20 expressed genes under ethanol-treated conditions of 1321DP8 (Table 4). The top 20 up-regulated expressed genes were mostly hypothetical proteins (7), transcript factors (TFs) (5), transporters (2), recombinase family protein, magnesium-protoporphyrin IX monomethyl ester (oxidative) cyclase, heme oxygenase (biliverdin-producing), acyltransferase family protein, tetratricopeptide repeat protein, and tRNA-Val. Several TFs or TF related genes such as HATPase_c (*sll0790*), Resolvase (*slr8029*), cNMP-binding (*slr0607*), Radical_SAM (*sll1876*), and DUF559 (*SGL_RS19135*).

**Table 4:** Top20 up-regulated and down-regulated DEGs in 1321DP8.

Regulation	Gene ID	log2FoldChange	Value of *p*	Gene description	TF family
Up-regulated	*slr5013*	6.948	0.000	Hypothetical protein	
	*slr8044*	5.862	0.023	Hypothetical protein	
	*slr0951*	5.862	0.023	2-C-methyl-D-erythritol 4-phosphate cytidylyltransferase	
	*sll0790*	5.644	0.041	HAMP domain-containing histidine kinase	HATPase_c
	*ST6803t05*	3.075	0.035	tRNA-Val	
	*sll1874*	2.912	0.000	Magnesium-protoporphyrin IX monomethyl ester (oxidative) cyclase	
	*sll1875*	2.849	0.000	Heme oxygenase (biliverdin-producing)	
	*slr1667*	2.696	0.000	Hypothetical protein	
	*sll1192*	2.545	0.003	Fluoride efflux transporter CrcB	
	*slr2103*	2.238	0.000	Acyltransferase Family protein	
	*SGL_RS20165*	2.234	0.030	Hypothetical protein	
	*sll1160*	2.128	0.044	Hypothetical protein	
	*slr1920*	1.975	0.014	Hypothetical protein	
	*slr8029*	1.958	0.025	Recombinase family protein	Resolvase
	*slr0607*	1.933	0.004	Cyclic nucleotide-binding domain-containing protein	cNMP_binding
	*slr1052*	1.908	0.001	Tetratricopeptide repeat protein	
	*slr1452*	1.895	0.000	Sulfate ABC transporter substrate-binding protein	
	*sll0419*	1.880	0.033	Hypothetical protein	
	*sll1876*	1.818	0.000	Oxygen-independent coproporphyrinogen III oxidase	Radical_SAM
	*SGL_RS19135*	1.806	0.000	DUF559 domain-containing protein	DUF559
Down-regulated	*slr5082*	−6.447	0.002	Rpn family recombination-promoting nuclease/putative transposase		
	*sll5062*	−6.447	0.002	Hypothetical protein		
	*slr6103*	−6.161	0.007	Protein of unknown function DUF45	DUF45	
	*sll1005*	−6.161	0.007	Nucleoside triphosphate pyrophosphohydrolase		
	*slr1148*	−6.161	0.007	DUF4269 domain-containing protein	DUF4269	
	*sll1319*	−5.804	0.023	DMT family transporter	DUF6	
	*SGL_RS20130*	−5.804	0.023	Hypothetical protein		
	*SGL_RS19080*	−5.586	0.041	Hypothetical protein		
	*SGL_RS19680*	−5.586	0.041	Hypothetical protein		
	*SGL_RS19235*	−5.586	0.041	Hypothetical protein		
	*sll0909*	−4.032	0.000	DnaJ family molecular chaperone		
	*SGL_RS20145*	−3.375	0.008	Hypothetical protein		
	*sll0494*	−3.375	0.008	Hypothetical protein		
	*SGL_RS19495*	−3.328	0.000	Hypothetical protein		
	*slr6031*	−3.251	0.013	DNA cytosine methyltransferase		
	*ssl7046*	−3.115	0.022	Putative toxin-antitoxin system toxin component		
	*sll8033*	−3.115	0.022	Hypothetical protein		
	*slr1170*	−2.840	0.000	TMEM165/GDT1 family protein		
	*slr0305*	−2.676	0.012	TVP38/TMEM64 family protein		
	*slr7071*	−2.570	0.018	CRISPR-associated endonuclease Cas1	

Top down-regulated genes included hypothetical proteins (9), TF or TF domain-containing proteins (DUF6, DUF45, DUF4269), DnaJ family molecular chaperone (*sll0909*), DNA cytosine methyltransferase, putative toxin-antitoxin system toxin component, TMEM family proteins (*slr1170* and *slr0305*), transposase and CRISPR-associated endonuclease Cas1 (*slr7071*). Compared with our previous OMIC studies, these TOP genes were not highlighted before (Wang et al., 2012; Zhu et al., 2015). Interestingly, we also found that most hypothetical proteins were on the top expressed gene list, suggesting their possible essential unknown protection functions (Wang et al., 2012).

DnaJ and DnaK belong to the heat shock protein family and are common stress responders. DnaJ family molecular chaperone (*sll0909*) was highly down-regulated in DP8 cells under ethanol stress. Similarly, we found a heat-shock DnaK homolog (*slr0086*) induced by ethanol stress (Wang et al., 2012). In this case, DP8 proteins may compensate for the defensive functions of DnaJ family proteins, at least at the transcriptional level.

It is well known that transcription factors (TFs) are involved in biofuel tolerance in bacteria (Ishihama, 2010; Fu et al., 2016). In cyanobacteria, a transcriptional regulator, *sll0794*, was involved in ethanol resistance directly, and the potential regulatory network mediated by *sll0794* was also proposed (Song et al., 2014). Through deletion mutagenesis, four TF, *slr0724*, *sll1392*, *sll1712*, and *sll0690*, were proven to be responsible for ethanol (Wang et al., 2012; Zhu et al., 2015) or butanol (Zhu et al., 2013) stress, respectively. In this study, unlike TFs mentioned above (*sll0794*, *slr0724*, *sll1392*, *sll1712*, and *sll0690*), significantly expressed novel TFs or TF-related genes, especially those TFs with unknown functions (DUF), were observed in 1321DP8, indicating CAHS might regulate biofuel stresses using different complicated pathways.

Strikingly, a toxin-antitoxin (TA) *ssl7046* was also highly down-regulated in DP8 cells. With no exactly known function, *ssl7046* was so far annotated as a ribonuclease. TA systems are involved in bacterial stress adaptation by regulating cell growth or death. They are abundantly existed in cyanobacteria but with little understanding (Fei et al., 2018). This is also the first report about TA systems involved in cyanobacteria against ethanol stress. Interestingly, CRISPR-associated endonuclease Cas1 (*slr7071*; Hein et al., 2013) was also showed up on the TOP down-regulated gene list. The links between the TA system and CRISPR are unclear; however, the expression of DP8 decreases the transcriptional levels of both TA and CRISPR systems in *Synechocystis*. Further investigations would be required to explore their connections.

## 4. Conclusion

In conclusion, we reported the hetero-expression of three CAHS proteins in a model cyanobacterium *Synechocystis*. TDP-expressed *Synechocystis* showed sensitive and tolerant phenotypes under different abiotic stresses. Notably, this analysis revealed that the induction of heat-shock protein and transporters, and modification of TCS, TA, and CRISPR systems were the central protection mechanisms against biofuel stress. The analysis provided a novel direction for engineering ethanol tolerance in cyanobacterium *Synechocystis*.

## Data availability statement

The datasets presented in this study can be found in online repositories. The names of the repository/repositories and accession number(s) can be found at: https://www.ncbi.nlm.nih.gov/, PRJNA888863.

## Author contributions

HZ and QYL performed the experiments and wrote the initial manuscript. QYL and BW designed the study. ZC and JW analyzed the data and revised the manuscript. All authors have read and approved the final manuscript.

## Funding

This research was supported by grants from the National Key R&D Program of China (2021YFA0910800, 2020YFA0908703, and 2018YFA0902500), and the National Natural Science Foundation of China (41876188).

## Conflict of interest

QL and BW were employed by Shenzhen Link Spider Technology Co., Ltd.

The remaining authors declare that the research was conducted in the absence of any commercial or financial relationships that could be construed as a potential conflict of interest.

## Publisher’s note

All claims expressed in this article are solely those of the authors and do not necessarily represent those of their affiliated organizations, or those of the publisher, the editors and the reviewers. Any product that may be evaluated in this article, or claim that may be made by its manufacturer, is not guaranteed or endorsed by the publisher.
