# Supplementary material for: Expression of tardigrade disordered proteins impacts the tolerance to biofuels in a model cyanobacterium Synechocystis sp. PCC 6803
Source: Front Microbiol. 2023 Jan 4;13:1091502. doi: 10.3389/fmicb.2022.1091502 (PMC9845703; doi:10.3389/fmicb.2022.1091502)
Supplement: Supplementary file 1 [file Data_Sheet_1.DOCX]

>CAHS106094

ATGGAGGCCATGAATATGAATATCCCCCGCGATGCCATGTTTGTTCCGCCACCGGAATCTGAGCAAAATGGGTATCATGAGAAGTCAGAAGTTCAGCAAACAAGTTATATGCAGAGTCAAGTCAAAGTGCCACATTATAATTTCCCGACACCATATTTTACGACTTCCTTTTCTGCGCAAGAGCTGCTGGGCGAAGGGTTTCAAGCCTCAATTTCCCGTATTTCAGCCGTTACGGAAGACATGCAGAGCATGGAAATCCCGGAGTTCGTTGAAGAGGCCCGCCGTGATTACGCAGCCAAAACACGTGAAAATGAGATGCTGGGGCAACAATATGAAAAAGAGCTGGAACGTAAGTCCGAAGCCTACCGCAAACATCAGGAAGTAGAGGCCGACAAAATCCGCAAAGAACTTGAAAAACAGCATATGCGTGATATTGAATTTCGGAAAGAAATTGCAGAACTGGCGATTGAGAACCAAAAACGTATGATCGATCTTGAATGCCGCTATGCAAAAAAAGACATGGACCGGGAACGCACAAAAGTTCGTATGATGCTGGAGCAACAGAAATTCCATAGTGATATCCAGGTAAATCTGGATTCTTCTGCGGCTGGGACCGAGAGCGGAGGTCATGTAGTGAGCCAGTCTGAAAAGTTCACCGAACGTAACCGCGAGATGAAACGCCACCACCACCACCACCACTGA

>CAHS77580

ATGAGTAACTATCAGCAAGAATCCAGCTATCAATATAGCGATCGCAGTAATAATGGCCAGCAACAAGAACAGCAAGAAAAGAAGGAAGTAGAACACTCGAGTTACACGCACACTGACGTAAAAGTAAATATGCCGAACTTGATTGCCCCTTTTATTTCATCTTCTGCGGGTCTGGCGCAGGAATTGGTCGGCGAGGGTTTCCAAGCAAGCGTATCTCGTATCACTGGCGCATCGGGCGAATTAACTGTGATTGATACTGAAGCAGAAACCGAGGAAGCTCGCCGTGACATGGAAGCCAAGGCACGTGAACAGGAGCTCCTGAGTCGTCAGTTTGAAAAAGAACTGGAACGTAAAACCGAGGCCTATCGCAAACAGCAGGAAGTTGAAACTGAAAAAATCCGCAAAGAATTAGAAAAACAGCATCTGCGCGATGTGGAGTTCCGTAAAGAACTCATGGAACAGACTATTGAAAACCAAAAACGCCAGATTGACCTCGAAGCGCGCTACGCTAAGAAAGAGCTCGAGCGTGAGCGGAATAAAGTCAAACGCGTTCTGGAACGTAGCAAATTTCATACAGACATCCAGGTAAACATGGAAGCGGCTGCGGGTTCTACTCACAGCGGCTCAAGCTCTGTGGCTGTTAGCGAAAGTGAAAAGTTCCAGACCAATAAC

>CAHS86272

ATGTCGCAGCAATATGAGAAGAAGGTTGAGCGGACGGAAGTCGTCTACGGAGGAGATCGTCGTGTCGAGGGCTCCGCGTCCGCATCCGCCGAGAAGACCACCAACTACACCCACACTGAGATCCGCGCTCCGATGGTGAATCCTCTGCCGCCGATCATTTCAACGGGTGCCGCTGGCCTGGCACAGGAGATTGTTGGGGAGGGCTTTACAGCCTCTGCCACACGTATATCCGGAGCTGCCGCCACCACCCAAGTCCTCGAATCTCAGGCATCCCGCGAACAGGCCTTCAAGGACCAGGAGAAGTACTCACGCGAGCAGGCCGCCATCGCCCGGGCGCACGACAAGGACCTTGAGAAGAAGACTGAGGAATATCGCAAGACTGCCGAGGCTGAAGCTGAAAAGATCCGCAAGGAGCTGGAGAAGCAACACGCCCGCGATGTGGAATTtCGCAAGGATCTCGTAGAATCCGCCATTGACCGCCAGAAACGGGAGGTGGATTTGGAAGCCAAGTACGCCAAGAAGGAGCTGGAACACGAGCGTGAACTGGCCATGAACGCGCTAGAGCAGTCGAAGATGGCCACCAATGTGCAAGTCCAAATGGACACCGCTGCTGGTACCACGGTCAGCGGAGGAACGACAGTCTCCGAACACACTGAAGTCCATGATGGGAAGGAGAAAAAAAGCCTCGGCGAGAAGATAAAGTCCCTTTTTCACCACCACCACCACCACTGA
